# A Word for the Medical Record

**Published:** 1876

**Authors:** A. G. Smythe

**Affiliations:** Baldwyn, Miss.


*A Word for the Southern Medical Record.*—As we enter upon a new year, permit me to say a word in behalf of THE SOUTHERN MEDICAL RECORD.

This journal, as is well known, is not published in the interest of any school, or for the benefit of any combination, party or faction, but solely for the benefit of the profession in the South and West. While it is not pretended that there is a paucity of medical literature in the land at present, the friends of THE RECORD are satisfied that it fills a place that is somewhat different from most of the medical journals of the day.

The pages of THE RECORD are open to all the legitimate members of the profession throughout the length and breadth of the land, so long as they are controlled by the Ethics of Medical law, or the rules of etiquette. Here all can have a hearing who are entitled to respect, without the danger of being frowned down, for fear of an expression of sentiment at war with the interests of the editors or publishers, who have no interest but honor, and the advancement of the medical science, at stake.

The whole editorial force—local and associate editors—together with the collaborators and contributors, will compare favorably with the staff of any journal in the country. True, they may not possess such an array of public teachers of distinguished characters, but in point of solid and sterling acquirements, hard labor, close application, and thorough practical observation, they will compare favorably with the same number of medical men in this or any other country.

The original communications are, generally, entitled to the highest regard, being based upon sound theory and of practical application, not encumbered with visionary or abstruse speculations.

The selections, from both foreign and domestic sources, are varied, practicable and well timed.

The communications, from the great mass of country practitioners, are brief, pointed and practical, generally free from any attempt to give fine specimen cases, partly or wholly manufactured from the whole cloth ; and such communications, if made, are sure to find a grave in the waste basket.

As to location, the SOUTHERN MEDICAL RECORD is published in the well-governed and prosperous State of Georgia; in the center of a wide field for the accumulation of medical knowledge and observation.

In conclusion, permit me to call the attention of the members of the medical profession, in the South and Southwest, to the necessity of supporting our own literature—not to the exclusion of literature from abroad, either foreign or domestic—but that we support our neighbors in all the material necessary to make up, establish, and support a good journal in our midst. It is as important that we are well informed as to what our neighbor over the way sees, knows, or does, as that we know what is being done in the remote portions of our own country, or in the great cities of New York, London, Paris, Berlin, or Vienna.

And now, doctors, one and all, who do not take the RECORD, do not say you are not able; if it is so that you are unable, why, quit practice at once, a business which will not support itself—abandon it: medical literature is an actual necessity to the practitioner. Do not say you are taking other journals, that is as it should be, but you need the SOUTHERN MEDICAL RECORD, and straightway enclose three dollars and your name to Dr. T. S. Powell, and you will not regret it.

A. G. SMYTHE.

BALDWYN, MISS., January 1st, 1876.

